# Implementation of a clinical breast exam and referral program in a rural district of Pakistan

**DOI:** 10.1186/s12913-024-11051-7

**Published:** 2024-05-10

**Authors:** Russell Seth Martins, Aiman Arif, Sahar Yameen, Shanila Noordin, Taleaa Masroor, Shah Muhammad, Mukhtiar Channa, Sajid Bashir Soofi, Abida K. Sattar

**Affiliations:** 1 Division of Thoracic Surgery, Department of Surgery, Hackensack Meridian School of Medicine, Hackensack Meridian Health Network, Nutley, NJ 08820 USA; 2 Aga Khan University, Karachi, 74800 Pakistan; 3 Department of Surgery, John Hopkins University, Baltimore, MD 21218 USA; 4 Department of Surgery, Link Building The Aga Khan University, Karachi, 74800 Pakistan

**Keywords:** Breast cancer, Clinical breast exam, Healthcare workers, Community outreach program

## Abstract

**Background:**

The role of clinical breast examination (CBE) for early detection of breast cancer is extremely important in lower-middle-income countries (LMICs) where access to breast imaging is limited. Our study aimed to describe the outcomes of a community outreach breast education, home CBE and referral program for early recognition of breast abnormalities and improvement of breast cancer awareness in a rural district of Pakistan.

**Methods:**

Eight health care workers (HCW) and a gynecologist were educated on basic breast cancer knowledge and trained to create breast cancer awareness and conduct CBE in the community. They were then deployed in the Dadu district of Pakistan where they carried out home visits to perform CBE in the community. Breast cancer awareness was assessed in the community using a standardized questionnaire and standard educational intervention was performed. Clinically detectable breast lesions were identified during home CBE and women were referred to the study gynecologist to confirm the presence of clinical abnormalities. Those confirmed to have clinical abnormalities were referred for imaging. Follow-up home visits were carried out to assess reasons for non-compliance in patients who did not follow-through with the gynecologist appointment or prescribed imaging and re-enforce the need for follow-up.

**Results:**

Basic breast cancer knowledge of HCWs and study gynecologist improved post-intervention. HCWs conducted home CBE in 8757 women. Of these, 149 were warranted a CBE by a physician (to avoid missing an abnormality), while 20 were found to have a definitive lump by HCWs, all were referred to the study gynecologist (CBE checkpoint). Only 50% (10/20) of those with a suspected lump complied with the referral to the gynecologist, where 90% concordance was found between their CBEs. Follow-up home visits were conducted in 119/169 non-compliant patients. Major reasons for non-compliance were a lack of understanding of the risks and financial constraints. A significant improvement was observed in the community’s breast cancer knowledge at the follow-up visits using the standardized post-test.

**Conclusions:**

Basic and focused education of HCWs can increase their knowledge and dispel myths. Hand-on structured training can enable HCWs to perform CBE. Community awareness is essential for patient compliance and for early-detection, diagnosis, and treatment.

**Supplementary Information:**

The online version contains supplementary material available at 10.1186/s12913-024-11051-7.

## Introduction

Breast cancer is the most frequently diagnosed malignancy (barring skin cancers) and the fifth leading cause of cancer-related deaths worldwide [1, 2]. According to GLOBOCAN 2022, 2.3 million new cases of breast cancer were diagnosed in 2022, with 666,103 patients dying from the disease [3]. Moreover, the incidence and mortality of breast cancer is expected to increase by 40% and 50% respectively by 2040 [3]. The rise in incidence is particularly steep in Asia, with these countries also seeing a significantly younger age of onset compared to the Western world [4, 5]. In Pakistan, one in every nine women suffers from breast cancer, with the country having one of the highest incidence rates in the region (around 2.5 times higher than neighboring countries such as Iran and India) [6, 7]. Breast cancer accounts for more than 20% of all malignancies in Pakistan, and almost half of all cancers in women [8]. 

The World Health Organization (WHO) has emphasized the role of early diagnosis of symptomatic breast cancer as a more feasible and economical strategy as compared to screening in resource-constrained countries [9, 10]. Screening for breast cancer allows for detection of breast cancer at an earlier stage (especially when small enough to remain undetectable on clinical examination) and leads to significantly better management outcomes and less treatment expenditure [1, 9]. While screening aims to identify lesions in asymptomatic and healthy individuals who have yet to develop clinical manifestations of disease, early detection of symptomatic breast cancer seeks to recognize individuals at an earlier stage than when they would otherwise present, allowing for more timely management and potentially better oncologic outcomes [9]. 

Early diagnosis and treatment are a cornerstone of efforts to reduce cancer-associated mortality in developed countries. In the United States (US), fewer than 20% of cancers present with advanced disease [11]. Data from Pakistan presents a stark contrast, with more than half of patients presenting with locally advanced or metastatic disease [11]. Mammography is the most effective screening modality for breast cancer in high-income countries. Multiple breast cancer screening trials have reported a reduction breast cancer-related mortality up to 25% among women undergoing mammography screening [12]. However, it remains under-utilized as a screening tool, both in developing and developed countries. Reasons for this range from misconceptions regarding screening methods, techniques, and radiation to lack of insurance or a care provider and fear of recall imaging, overdiagnosis leading to unnecessary biopsies and treatment and side effects [13, 14]. In the US, more than 75% of eligible women are screened for breast cancer via mammography [15]. In a lower-middle-income country (LMIC) like Pakistan, access to investigations such as mammogram, breast ultrasound and needle biopsy is limited due to lack of availability of machines and trained personnel, lack of awareness and financial limitations (75% of healthcare financing in Pakistan is out-of-pocket and over one-third of the population lives below the poverty line) [16, 17]. In addition, conservative sociocultural norms and religious factors also prevent women from seeking routine healthcare [18]. Given the lack of healthcare access coupled with a largely conservative culture, community outreach programs with home visits may be the ideal system for bringing initial breast cancer recognition home to the rural communities, enabling early confirmation of disease and initiation of treatment. Similar outreach programs have met with considerable success in other aspects of healthcare. These include programs improving screening and prevention of malaria, tuberculosis, and HIV and those targeting improvement maternal and neonatal mortality [19, 20]. Thus, screening, and early detection interventions implemented in LMICs like Pakistan must take into account the local healthcare systems and social structures.

Clinical breast examination (CBE) is recommended as the preferred approach for early detection of symptomatic and clinically detectable breast cancer in LMICs such as Pakistan. It consists of inspection and palpation of the breasts and regional (axillary, supraclavicular, infraclavicular and cervical) lymph nodes of the patient in a sitting and supine position [21]. It can be readily performed by a primary care physician to identify abnormal breast findings and determine the need for further evaluation.[22], [23] In fact, while mammography is expected to miss over 20% of breast cancers, CBE is able to detect 3–45% of these false negative cases [24–26]. 

Due to the aforementioned sociocultural barriers towards mammographic breast screening in Pakistan, it is vital that early detection interventions employ more feasible methods such as CBE. Thus, the objective of this study was to describe the outcomes of a community outreach breast education, home CBE and referral program for early recognition of breast abnormalities and improvement of breast cancer awareness in a rural district of Pakistan. We conducted a community outreach and referral program where home CBE visits were conducted by trained healthcare workers (HCWs) for early detection of breast signs and symptoms, in a rural district of Pakistan. Women who had clinical abnormalities detected upon examination were then referred for further evaluation. During these visits, the women were also educated regarding breast cancer management. In this study, we report our results and experiences with this program. We believe that it is important to reinforce that early detection interventions for breast cancer may be implemented in LMICs like Pakistan using CBE as the preferred approach. Given that most patients with breast cancer present with advanced disease, CBE may be able to identify characteristic breast changes earlier and allow for timely treatment of the tumor at earlier stages [27]. 

## Materials and methods

### Study design and setting

A quasi-experimental study was carried out over September 2021 - September 2022 in Sindh, Pakistan. The study team was primarily based at Aga Khan University (AKU) in Karachi, Sindh, while the field location where the community outreach program was implemented was situated in the Dadu district of Sindh, Pakistan. The Aga Khan University is an academic tertiary care private hospital and a health services agency of the Aga Khan Development Network (AKDN) in Pakistan. This study featured a collaboration between the Departments of Surgery and Maternal and Child Health at AKU. Ethical approval was obtained from the ethical review committee at AKU.

The Dadu district covers 19,070 km^2^ in interior Sindh and is divided into four sub-divisions which are further divided into Union Councils (UC). The UC is the smallest administrative unit of Pakistan. The field location of our study consisted of five UCs within the Johi subdivision of the Dadu district. Some census data of the five included UCs, as collected by the AKU for local projects, are shown in Table 1. Approximately 48.7% of the population is female. As of 2021, it has 47 Basic Health Units (BHU) and 5 Rural Health Centers (RHC) with a total of 503 beds (BHUs and RHCs are first-level primary healthcare facilities that serve rural populations). The doctor -to-patient ratio is 1:6,030, nurse-to-patient ratio is 1:39,629, and bed-to-patient ratio is 1:3,309 [28]. 

**Table 1:** Census Data for the UCs in the Johi subdivision of Dadu, Pakistan

Target UCs	Households (number of households)	Population
Johi	2,808	18,283
Johi Town UC-1	1,429	11,317
Johi Town UC-2	1,389	11,919
Kamal Khan	1,403	10,284
Peer Mashaikh	1,628	12,220
Total	8,657	64,023

### Study population and sample size calculation

The total population within the five target UCs was 64,023. Our target sub-population consisted of all adult women ≥ 18 years of age. Using an estimated 20% prevalence of abnormal CBE according to a similar study conducted in Tajikistan [29], 80% power, 95% confidence level, and design effect of 2, we calculated the minimum required sample size to be 547 individuals. This was inflated by 100% to mitigate against extreme rates of individuals being lost to follow-up, which we anticipated to be a significant real-world challenge, yielding a final minimum required sample size of 1,094. Cluster convenience sampling was used to identify women in the community.

### Training workshop and outreach program

The study schema consisted of the following interventions in sequence as described:

i. *Training of Health Care Workers (HCWs)*: Non-physician HCWs received training at AKU, Karachi in September – October 2021. This specialized training program was designed to enhance HCWs’ skills in identifying suspicious breast problems, making appropriate and timely referrals, and improving general knowledge regarding breast cancer. This training was conducted and overseen by an attending breast surgeon at AKU. HCWs were taught how to perform clinical breast examinations (CBEs) and engaged in hands-on practice sessions with simulated breast disease models and real patients in clinics. In addition, the HCWs were educated regarding general knowledge regarding breast cancer, with special emphasis on treatment, evaluation and commonly held misconceptions among the public. Pre and post-intervention surveys were administered to evaluate improvement in knowledge.
ii. *Community outreach program with home visits*: The HCWs were deployed into the community in the Johi subdivision in October 2021. The initial series of home visits took place between October 2021 to February 2022, with the HCWs performing home visits in groups of two. Each visit began with an introductory and informed consent-seeking debriefing, followed by CBE of all consenting adult women belonging to a household, an assessment of baseline breast cancer-related knowledge, and lastly, a brief, standardized educational intervention delivered verbally (Supplement). For each CBE performed, a checklist of examination findings was completed. In the event of any abnormal finding, a referral to a local gynecologist within Johi was made. All interactions during the home visits were conducted in the Sindhi language, which is the native language of the region.
iii. *Visit to the local gynecologist*: Patients who complied with their referral (for a palpable breast concern) were evaluated by a gynecologist at the local District Health Quarter. The gynecologist repeated a CBE on all referred patients in order to validate the HCWs’ examination findings. All eligible patients were then referred for breast imaging, either mammography or ultrasound, to the nearest facility within Johi.
iv. *Follow-up home visits*: The HCWs attempted to conduct follow-up home visits for all women who were non-compliant with initial referral to a gynecologist. These follow-up visits took place six months after the initial series of home visits. Patients were questioned as to the reasons for their non-compliance with referral using a self-designed structured questionnaire (Supplement: Sect. 4). In addition, the breast cancer-related knowledge survey was re-administered to the women to gauge improvement in knowledge since the educational intervention delivered at the initial home visits. Finally, the importance of complying with referral for future evaluation, diagnosis and management was re-emphasized to all patients.

*Validation of Data Collection Tools*:

i. *CBE checklist*: This was a self-designed checklist (Supplement: Sect. 3) that included all the important components of a CBE, including a brief history of relevant symptoms (pain, discharge), breast inspection (skin changes, or changes in breast size, shape, or symmetry, and nipple changes), and breast palpation (presence of lumps in the axilla or breast).
ii. *Breast cancer-related knowledge survey*: Separate surveys were administered to the HCWs and the women within the general community (Supplement: Sects. 2 and 3). Both surveys were designed by faculty within the Section of Breast Surgery at AKU. Prior to its use, the survey for women within the community was pretested amongst 30 local women for content, comprehensibility, and language. Minor adjustments were made on the basis of this pilot procedure.

### Statistical analysis

All analyses were performed using SPSS (Statistical Package for Social Sciences) version 23.0 (IBM Corporation, Armonk, New York). Descriptive analysis was performed whereby categorical values were reported using frequencies and percentages. McNemar’s test was used to compare changes in knowledge across the multiple administrations of the breast cancer-related knowledge surveys. A p-value less than 0.05 was considered significant for all the analysis.

## Results

### Education and training of the HCWs

A total of 8 HCWs were trained. Tables 2 and 3 show the changes in breast cancer-related knowledge after the educational and training intervention for the HCWs. The absolute percentage increase in HCWs who correctly believed that breast cancer can occur in men, and in women despite breast feeding their children, was 50%. In addition, the percentage of respondents who believed that women with a painless lump should visit a healthcare professional increased from 87.5 to 100%. The absolute percentage of HCWs who correctly identified painless lump and bloody nipple discharge as a symptom suspicious of breast cancer increased by 12.5% and those that identified dimpling of skin as a suspicious symptom increased by 25%. The percentage of HCWs who correctly believed that a tissue biopsy could be used to diagnose breast cancer increased from 62.5 to 87.5%.

**Table 2:** Healthcare workers’ general knowledge regarding breast cancer

Statement/Question	Pre-Intervention; *N* (%)	Post-Intervention; *N* (%)
**“Breast cancer occurs in women only”**		
Yes	5 (62.5)	0 (0)
No*	3 (37.5)	8 (100)
**“Breast cancer can also occur in men.”**		
Yes*	4 (50)	8 (100)
No	4 (50)	0 (0)
**“Can breast cancer occur in mothers who breastfed?”**		
Yes*	4 (50)	8 (100)
No	4 (50)	0 (0)
**“Can breast cancer occur without a family history?”**		
Yes*	7 (87.5)	6 (75)
No	1 (12.5)	2 (25)
**“Most cases of breast cancer are due to:”**		
No identifiable cause/sporadic*	6 (75)	7 (87.5)
Family history/germline mutations	1 (12.5)	1 (12.5)
Do not know	1 (12.5)	0 (0)
**“Breast cancer is a contagious disease.”**		
Yes	0	0
No*	8 (100)	8 (100)
**Should a woman with a painless lump be encouraged to consult a healthcare professional?**		
Yes*	7 (87.5)	8 (100)
No	1 (12.5)	0 (0)

* Denotes the correct response

**Table 3:** Healthcare workers’ diagnosis- & management-related knowledge regarding breast cancer

Statement/Question	Pre-Intervention; *N* (%)	Post-Intervention; *N* (%)
**“Which of these are potentially signs of breast cancer?”**		
Lump with Pain	6 (75)	5 (62.5)
Lump without Pain*	7 (87.5)	8 (100)
Bloody discharge from nipple*	7 (87.5)	8 (100)
Dimpling of skin*	6 (75)	8 (100)
Any unusual change in the shape of breast(s)*	5 (62.5)	4 (50)
Thickening/ulceration*	3 (37.5)	5 (62.5)
**A breast lump in a woman is always a cancer.**		
Yes	0	0
No*	8 (100)	8 (100)
**“What is the next step after you identify a patient with a new breast lump?”**		
Wait and watch.	1 (12.5)	0 (0)
Refer the patient for imaging *	5 (62.5)	6 (75)
Refer the patient directly for surgery	0 (0)	2 (25)
Do nothing	1 (12.5)	0 (0)
Don’t know	1 (12.5)	0 (0)
**Can breast cancer be diagnosed by clinical breast examination?**		
Yes	7 (87.5)	5 (62.5)
No*	1 (12.5)	3 (37.5)
**Can breast cancer be diagnosed by imaging (mammography/ultrasound)?**		
Yes	6 (75)	8 (100)
No*	2 (25)	0 (0)
**Can breast cancer be diagnosed by biopsy?**		
Yes*	5 (62.5)	7 (87.5)
No	3 (37.5)	1 (12.5)
**“Can needle biopsy of a potentially cancerous breast lump can lead to spread of cancer?”**		
Yes	1 (12.5)	0 (0)
No*	7 (87.5)	8 (100)
**“Is breast cancer treatable?”**		
Yes*	7 (87.5)	8 (100)
No	1 (12.5)	0 (0)

* Denotes the correct response

### Implementation of the outreach program

**Fig. 1:**
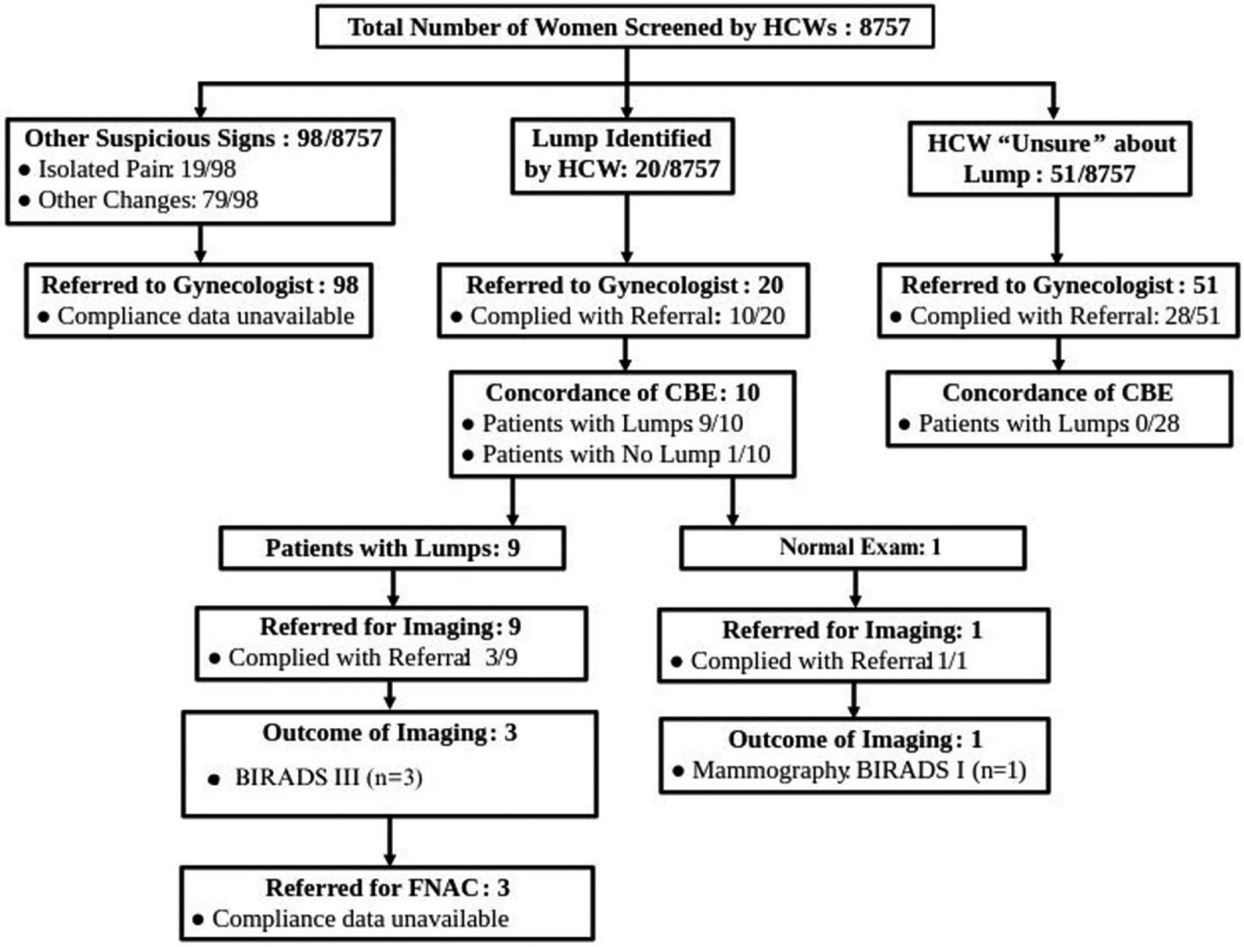
Outcomes of community outreach Breast referral program. HCW: Health Care Workers; CBE: Clinical Breast Examination; FNAC: Fine Needle Aspiration Cytology

A total of 8,757 women were screened by the HCWs in the field during initial series of home visits. A palpable breast lump was identified in 20/8,757 women, while other palpable or visible breast concerns warranting further evaluation were identified in 98/8,757 women. In addition, HCWs were unsure about the presence of a lump in 51/8,757 women. Keeping a low threshold for seeking a physician’s evaluation and prompt referrals, these 169/8,757 patients were all referred to a gynecologist for further examination. However, only 38/169 patients (ten with a palpable breast lump and 28 for which the HCWs exercised caution-either noted other breast concerns or were unsure) complied with initial referral to the gynecologist. Out of the 28 patients (where HCWs noted breast concerns or were unsure about a lump), none were found to have a lump on the gynecologist’s CBE examination. Out of the ten patients in which the HCWs had positively identified the breast lumps, nine patients (90% concordance) were confirmed to have a breast lump on the gynecologist’s CBE. However, all these ten patients were referred for imaging with only 4 of them complying. Amongst these 4 patients who had breast imaging, one patient had BI-RADS (Breast Imaging Reporting and Data System) category I finding (i.e. negative imaging) and 3 patients had BI-RADS category III findings (Lump with extremely low probability of malignancy). The outcomes of the CBE and referral program are illustrated in Fig. 1.

At the follow-up home visits to the 131 patients who had been non-compliant with initial referral, the most common reasons for non-compliance were assessed by the HCWs **(**Table 4). The most common reasons for non-compliance were a belief that follow-up was not important (42.0%), lack of money to visit the gynecologist (24.4%), not having anyone to accompany them (9.2%), long distance to travel for the appointment (7.6%).

**Table 4:** Reasons for Non-Compliance to Referral (patients with non-compliance/whose referral compliance data was unavailable)

Variable/Question	*N* (%)
**Follow-up home visit possible?**	
Yes	119 (90.8)
No	12 (9.2)
**Reason why follow-up home visit was not possible.**	**N = 12**
Migrated	11 (91.7)
Died	1 (8.3)
**Were you aware that you needed to visit the gynecologist?**	**N = 119**
Yes	98 (82.4)
No	21 (17.6)
**Reasons for non-compliance**	**N = 119**
I did not come for the follow-up visit because I felt it was not important	50 (42.0)
I did not have the money to pay for the follow-up visit	29 (24.4)
I was not able to come as I did not have anyone to accompany me	11 (9.2)
The distance to travel for the appointment was too far	9 (7.6)
I did not come for the follow-up visit because I forgot about it	9 (7.6)
I did not have transport available to come for the follow-up visit	7 (5.9)
I was not able to come due to household responsibilities	0 (0)
I was not able to come as I have a child/children to care for	7 (5.9)
I was not able to come as I have elderly people in my house, who I care for	0 (0)
I was prohibited to come for the follow-up visit by a family member(s)	4 (3.4)
I was not satisfied with my experience at the previous appointment	0 (0)
I visited a different healthcare facility for a follow-up	0 (0)
I did not feel well enough to come for the follow up appointment	0 (0)
Other (please specify)	6 (5.0)

### Increase in community awareness regarding breast cancer

A comparison of the women’s knowledge regarding breast cancer at the time of initial visit and later at follow-up is shown in Table 5. The percentage of women who had heard of breast cancer increased from 54.6 to 100%, the percentage of women who were aware that breast cancer was treatable increased from 32.8 to 61.3%. The percentage of women understood the need to consult a healthcare professional upon finding a lump increased from 50.4 to 94.1%.

**Table 5:** Community’s general knowledge regarding breast cancer

Statement/Question	Initial	Follow-Up	*P*-Value
**Have you ever heard of breast cancer?**			<0.001
Yes	65 (54.6)	119 (100)	
No	54 (45.4)	0 (0)	
**Is breast cancer treatable?**			**< 0.001**
Yes *	39 (32.8)	73 (61.3)	
No	16 (13.4)	30 (25.2)	
Don’t Know	64 (53.8)	16 (13.4)	
**Is breast cancer contagious?**			**0.012**
No *	18 (15.1)	34 (28.6)	
Yes	34 (28.6)	42 (35.3)	
Don’t Know	67 (56.3)	43 (36.1)	
**Which of the following signs/symptoms can raise concerns for breast cancer?**			
Breast Lump *	53 (44.5)	104 (87.4)	**< 0**.**001**
Bloody Discharge from Nipple *	13 (10.9)	8 (6.7)	0.359
Dimpling of Skin overlying Breast *	4 (3.4)	1 (0.8)	0.375
Unusual Change in Shape/Size of Breast *	6 (5.0)	1 (0.8)	0.125
Thickening of Skin overlying Breast *	11 (9.2)	7 (5.9)	0.454
**Can breast cancer occur in men?**			**< 0.001**
Yes *	21 (17.6)	65 (54.6)	
No	92 (77.3)	22 (18.5)	
Don’t Know	6 (5.0)	32 (26.9)	
**Can breast cancer occur in the absence of a family history?**			**< 0.001**
Yes *	47 (39.5)	59 (46.9)	
No	66 (55.5)	15 (12.6)	
Don’t Know	6 (5.0)	45 (37.8)	
**Can breast cancer occur even if a woman has breastfed her child?**			**< 0.001**
Yes *	49 (41.2)	91 (76.5)	
No	64 (53.8)	7 (5.9)	
Don’t Know	6 (5.0)	21 (71.6)	
**If a woman has a painless lump in her breast, should she consult a health care professional?**			**< 0.001**
Yes*	60 (50.4)	112 (94.1)	
No	2 (1.7)	2 (1.7)	
Don’t Know	57 (47.9)	5 (4.2)	

* Denotes the correct response

## Discussion

The purpose of this study was to evaluate the feasibility of the real-world implementation of a large-scale clinical breast examination and referral community outreach program in a rural district of Pakistan. Secondarily, we also explored the feasibility of delivering basic breast cancer-related knowledge to the community via non-physician HCWs. This program was the first of its kind for breast cancer detection in the country. The key positive takeaways from our experience were that it is: (i) possible to train non-physician HCWs to perform a comprehensive CBE and identify examination findings warranting referral and further evaluation, (ii) practically feasible to implement a large-scale community outreach program with home-visits for mass detection of breast cancer, (iii) possible to increase community knowledge and awareness for breast cancer by imparting education at the home-visits when CBE was performed. However, we encountered several real-world challenges that precluded the full realization of this outreach program’s impact. Only 50% of women initially identified by the HCWs as having a breast lump followed through with referral to the gynecologist, and only 40% of women followed up with subsequent referral for imaging. None of the patients eventually referred for histopathological evaluation ended up complying with the referral. However, prior experience with a similar program by the AKDN in Tajikistan demonstrated that with appropriate follow-ups, breast cancer may be detected in up to 0.2% of the women in the community [29]. Although this rate is slightly lower than the reported incidences of mammographic screening-detected breast cancers in the literature (0.5–0.8%), it underscores the potential for success of CBE-based programs as an early detection strategy in low-resource communities [30, 31].

### Accuracy of CBE by non-physician HCWs and effectiveness of educational interventions

Overall, the theoretical frameworks and foundations of this large-scale clinical breast examination and referral community outreach program were observed to be largely successful. We were able to achieve a high degree of concordance (90%) between the CBE findings of the HCWs and the gynecologist, indicating that it is possible and feasible to leverage HCWs for the early detection of symptomatic breast cancer. Another study carried out in Malawi to train community laywomen to conduct CBE in the community showed 88% concordance between CBE performed by the HCWs and those performed by the physicians [32]. This is exceedingly important in a LMIC like Pakistan, where the ratio of physicians to population is a major impediment to healthcare access. In Pakistan, there are only 170,000 general practitioners to serve a population of over 230 million individuals. Thus, a major bottleneck for the delivery of high quality breast cancer-related healthcare is the timely initial identification of these patients from the community. Utilizing existing community outreach frameworks, such as the Lady Health Worker (LHW) Program, cite which was in Pakistan in 1994 [33, 34]. While the LHW Program was initially developed for promoting family planning and maternal health, the model has been adapted for other major public health interventions such as immunizations and basic preventative healthcare. These LHWs are salaried and recognized as part of the healthcare workforce. Since LHWs are recruited from within the community itself, one of the major strengths of such a program is their ability to deliver culturally appropriate healthcare to populations with limited access to healthcare facilities. Thus, based on the successful training of HCWs in our study, we believe that the LHW Program model can be effectively adapted for the early recognition of breast abnormalities in women who would otherwise go undetected. However, it is important to know that the training of the HCWs in our study was performed by a fellowship-trained breast surgeon at a tertiary care hospital in one of the major cities of Pakistan. To ensure the feasibility, uptake, and growth of our model throughout the underserved regions of the country, it is important that a certain degree of sustainability is achieved. In future iterations of this model, we plan to assess the effectiveness of cascade learning with peer-to-peer teaching. In such a model, HCWs initially trained by a breast surgeon will subsequently assume the role of trainers themselves and teach other HCWs/LHWs how to perform a CBE. Interestingly, the study conducted in Malawi trained non-HCWs to serve as “Breast Health Workers”, highlighting the potential to leverage non-HCW professionals to perform a health-related role in communities with low HCW-to-patient ratios [32]. 

### Community education and awareness

Despite the successful and rigorous implementation of the CBE and referral community outreach program, the Achilles’ heel of this project was the pervasive lack of community awareness regarding the importance of following up with referrals. This was compounded by other sociocultural barriers such as financial constraints, transportation issues, and a lack of family support to visit the healthcare facility. Thus, it is important that future iterations of similar public health interventions be cognizant of these challenges and seek to mitigate them to the best of their ability. Indeed, the most modifiable of these obstacles is the lack of awareness which can be countered by greater community education during home visits, with a particular focus on emphasizing the potential consequences of non-compliance with diagnostic evaluations. Our results demonstrated the feasibility of educating HCWs to subsequently serve as teachers for the community, and that the newly gained knowledge remained reasonably intact even at a follow-up of six months. A study in Vietnam showed that repeated breast cancer-related educational interventions were successful in increasing compliance with referrals for breast cancer evaluation. [35] In addition, a more robust follow-up system including frequent interaction and monitoring of patients could help boost compliance with referrals and better continuity of care. For example, routinely scheduled phone calls could be made to the patient as a reminder to follow-up with their referrals. Moreover, for women who are not able to comply with their referrals because of the absence of a family member to accompany them, arrangements may be made whereby LHWs could accompany them as their attendants.

The other challenges, however, harken to well-known and longstanding problems with the healthcare system in Pakistan, where most of the population is unable to afford even basic healthcare. In such a setting, Universal Healthcare Coverage (UHC) emerges as the only viable solution to the masses. An attempt at such a system, the Sehat Sahulat Program (SSP; translates to Health Facility Program) was introduced in 2016 by the provincial government of Khyber Pakhtunkhwa, one of the five provinces of Pakistan [36]. The SSP was designed to cover a broad range of health conditions and services, including breast cancer diagnosis and evaluation. While the program was met with success in its initial years, and even expanded into some of the other provinces, instability in the political and economic infrastructures of Pakistan have limited its growth, uptake, and effectiveness. Ideally, mass community interventions for early breast cancer detection such as ours could be integrated with UHC programs such as SSP to ensure patient compliance, continuity of care, and maximization of invested resources most effectively.

### Limitations

Our study has several limitations that we would like to acknowledge. Firstly, we were unable to calculate a study participation rate as the HCWs did not record the number of informed refusals that they received from women in the community. Secondly, as mentioned earlier, compliance with referrals was exceedingly poor and limited the realization of the true impact of the program. Thirdly, given the limited number of HCWs included, we were unable to perform statistical comparisons to evaluate the improvement in HCWs knowledge. Lastly, the evaluation of the long-term impact and sustainability of the program was limited, presumably due to the influence of sociocultural barriers on the health-seeking behaviors of the women.

## Conclusion

This study describes the real-world implementation of a large-scale clinical breast examination and referral community outreach program in a rural district of Pakistan. Our study highlights the importance of CBE programs in early recognition of breast abnormalities/lumps, in regions where mammography is not feasible. Such training programs may lay the foundation for improved provider and community awareness, and examination at the patient’s doorstep and initiate referrals. However, for such programs to ultimately lead to earlier detection of breast cancer/downstaging of disease, community awareness and political buy-in from governmental stakeholders would be critical. Lastly, for such a program to have a truly national impact and be sustainable, more widespread training of HCWs using cascade learning and peer-to-peer teaching models would be necessary.

### Electronic supplementary material

Below is the link to the electronic supplementary material.

Supplementary Material 1

## Data Availability

The datasets generated and/or analysed during the current study are not publicly available since the Ethical Review Committee guidelines does not allow institutional data to be dispersed. However, the data is available on reasonable request to the corresponding author.

## Abbreviations

GLOBOCAN: Global Cancer Observatory
US: United States
LMIC: Lower-Middle-Income Country
WHO: World Health Organization
CBE: Clinical Breast Examination
AKU: Aga Khan University
UC: Union Council
HCW: Healthcare Workers
BI-RADS: Breast Imaging Reporting and Data System
LHW: Lady Health Worker
UHC: Universal Health Coverage
