# Supplementary material for: Implementation of a clinical breast exam and referral program in a rural district of Pakistan
Source: BMC Health Serv Res. 2024 May 10;24:616. doi: 10.1186/s12913-024-11051-7 (PMC11083956; doi:10.1186/s12913-024-11051-7)
Supplement: Supplementary file 1 — Supplementary Material 1 [file 12913_2024_11051_MOESM1_ESM.pdf]

Supplement

S. No.	Section	Page Number
1	Section 1	2
2	Section 2	3-5
3	Section 3	6-7
4	Section 4	8

Section 1

Checklist For Breast Examination

Section Status: 1. Completed 2. Partially Completed 3. Refused 4. Write reason for option 2 & 3: ____________________________

1. Ask about any painful area in the breast, armpit, or surrounding area.
   a. Pain present Yes/No
      i. If yes, specify the area: _______________________

2. Ask about any **spontaneous** nipple discharge.
   a. Discharge present Yes/No

3. Observe (Inspection) comparing both breasts:
   a. Skin changes:
      i. Changes in overlying skin such as: Yes/No
         1. Redness Yes/ No
         2. Dimpling/ulcer Yes/ No
         3. Skin thickening Yes/ No
      ii. Swelling/visible lump Yes/ No
      iii. Others, specify: _______________________________

   b. Compare the shape and size of both the breasts.
      i. Normal bilaterally Yes/ No
      ii. Abnormal Yes/ No
         Specify: __________________________________

   c. Observe nipples of both breasts.
      i. Retraction (Right/Left/Both)
      ii. Crusting (Right/Left/Both)
      iii. Normal (Right/Left/Both)

   d. Any lump felt/palpated in armpit. Yes/ No
   e. Any lump felt/palpated above the collar bone. Yes/ No
4. Palpation (palpate both the breasts up to the collar bone and mid axillary lines to identify any abnormality/lump).
   a. Any swelling/lump identified Yes/ No
      i. Specify the area(s): _______________________________

5. Any other relevant examination findings, please specify: ________________________

Section-2

Knowledge and Attitudes Regarding Breast Cancer in the Healthcare Workers (HCW)

1. Breast cancer only occurs in women.
    a. Yes
    b. No

2. Can Breast cancer occur in men?
    a. Yes
    b. No

3. Can a woman who has given birth to a child and breast-fed, get breast cancer?
    a. Yes
    b. No

4. Can a woman who does not have a family history of breast cancer get diagnosed with breast cancer?
    a. Yes
    b. No

5. Most breast cancers are, choose the correct answer:
    a. Associated with a family history.
    b. No clearly identifiable cause

6. Which of the following presentations may be suggestive of breast cancer? (choose all that apply)
    a. Lump with pain
    b. Lump without pain
    c. Bloody discharge from nipple
    d. Dimpling of skin
    e. Any unusual change in the shape of breast(s)
    f. Thickening/ulceration of skin
    g. Don't know
    h. Others (specify): ---

7. Which of the following modalities are needed to diagnose breast cancer? (Choose all that apply)
    a. By checkup/examination
    b. By mammography
    c. By ultrasound
    d. By biopsy
    e. Don't know
    f. Others (specify): ---

8. What is the next step after a woman has been found with a new palpable mass in the breast on your exam?
    a. Refer the patient for breast imaging such as mammogram/ultrasound/biopsy.
    c. Refer the patient directly for breast surgery.
    d. Wait and watch.
    e. Don't know

9. Needle biopsy can lead to spread of cancer.
    a. Yes
    b. No

10. Does breast cancer spread from person to person?
    a. Yes
    b. No

11. A breast lump in a woman is always a cancer.
    a. Yes
    b. No

12. It is best that Breast cancer left untouched.
    a. Yes
    b. No

13: In your opinion, is breast cancer treatable?
    a. Yes
    b. No
    c. Don't know

14. If a woman has a painless lump in her breast, in your opinion, should she consult a health care professional?
    a. Yes
    b. No

15. Match the correct items:

**Changes in breasts**	**Picture no.**
Lump in breast	
Abnormal changes in nipple	
Dimpling of skin	
Bloody discharge from nipple	
Any unusual change in the shape of both breast (s)	
Thickening/ulceration of skin	

A **B** **C**

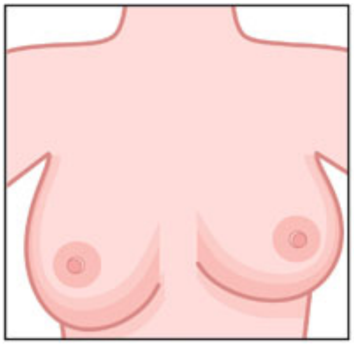

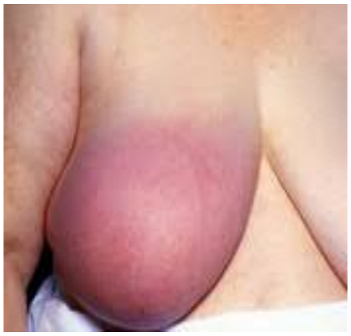

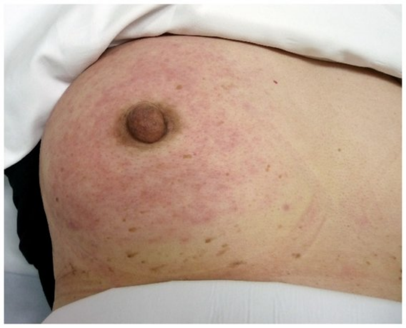

D **E** **F**

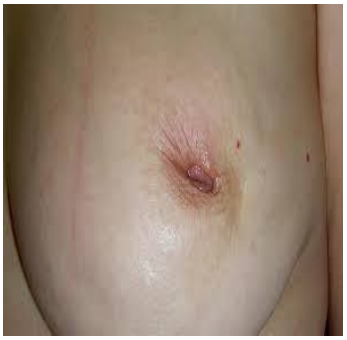
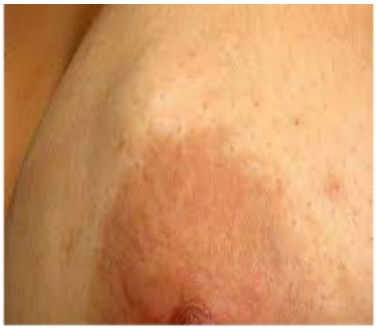
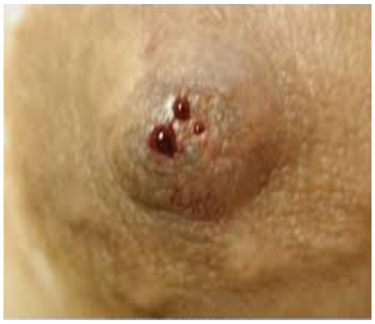

Section-3

Knowledge and Attitudes Regarding Breast Cancer in the Community

Components of the Educational Intervention

The following topics were covered in an informal, semi-structured discussion with learners:

- An overview of breast cancer including its incidence and risk factors.
- Signs and symptoms of breast cancer
- Detection, evaluation, and diagnosis of breast cancer (importance of clinical breast exam, mammography, ultrasound and biopsy)
- How to conduct a self-breast examination.
- What steps to take when an individual feels a lump in their breast.
- What are the treatment options if breast cancer is diagnosed.

Questionnaire

Name of the respondent: ____

Age/Date of birth: ________________

Woman's ID: ________________________ Household ID: ___________

Date of Visit: ________________________ Union Council: __________________

Village/Ward name: __________ Village/Ward code: ____________

Section Status: 1. Completed 2. Partially Completed 3. Refused 4. Write reason for option 2 & 3: ____________________________

1. Have you ever heard of Breast cancer?
   a. Yes b. No c. Don't know

2. In your opinion, is breast cancer treatable?
   a. Yes b. No c. Don't know

3. Does breast cancer spread from person to person by contact (contagious)?
   a. Yes b. No c. Don't know

4. Do you know anyone who has been treated for breast cancer?
   a. Yes b. No c. Don't know

5. In your opinion, which of the following may raise concerns about breast cancer? (Choose all that apply)
   a. Lump
   b. Blood discharge from nipple
   c. Dimpling of skin
   d. Any unusual change in the shape of breast(s)
   e. Thickening of skin
   f. Don't know
   g. Others (specify): --

6. Can Breast cancer occur in men?
   a. Yes b. No c. Don't know

7. Can somebody get breast cancer even if none of their family members have been diagnosed with breast cancer?
   a. Yes b. No c. Don't know

8. Can a woman get breast cancer even if she has nursed a child?

a. Yes b. No c. Don't know

9. In your opinion, how can breast cancer be diagnosed? (Choose all that apply
   a. By checkup/examination
   b. By mammography
   c. By ultrasound
   d. A combination of checkup/tests and biopsy
   e. Don't know
   f. Others (specify): --

10. If a woman has a painless lump in her breast, in your opinion, should she consult a health care professional?
    a. Yes b. No c. Don't know

11. At this point in time, do you have any lump or breast abnormality that you can see or feel?
    a. Yes b. No c. Don't know
    If yes, what signs are present? --

12. Will it be feasible for you if a health worker comes to your place and teaches you how to examine your breast?
    a. Yes b. No c. Don't know

13. If you are asked to carry out breast self-examination on a monthly basis, will you be able to do this?
    a. Yes b. No c. Don't know

Section 4

Reasons for Loss-to-Follow-Up for Last Appointment

Tick all that apply as reasons for not attending the last scheduled appointment

- Lack of adequate transportation facilities
- Long distance.
- Financial constraints.
- Household responsibilities.
- Childcare
- Care for elderly
- Prohibited by family member(s)
- No attendant/nobody available to accompany patient
- Forgot about appointment
- Were not properly informed about their appointment
- Thought appointment was not important
- Not satisfied with previous appointment
- Visited other healthcare facility
- Not feeling well enough to come to appointment
- Other (please specify):

  ____

  ____
